# Mitochondrial Ca^2+^ uniporter haploinsufficiency enhances long-term potentiation at hippocampal mossy fibre synapses

**DOI:** 10.1242/jcs.259823

**Published:** 2022-11-23

**Authors:** Michael J. Devine, Blanka R. Szulc, Jack H. Howden, Guillermo López-Doménech, Arnaud Ruiz, Josef T. Kittler

**Affiliations:** ^1^ Department of Neuroscience, Physiology and Pharmacology, University College London, Gower Street, London WC1E 6BT, UK; ^2^ Department of Pharmacology, School of Pharmacy, University College London, Brunswick Square, London WC1N 1AX, UK

**Keywords:** Mitochondria, Ca^2+^, MCU, Long-term potentiation, Mossy fibre synapse

## Abstract

Long-term changes in synaptic strength form the basis of learning and memory. These changes rely upon energy-demanding mechanisms, which are regulated by local Ca^2+^ signalling. Mitochondria are optimised for providing energy and buffering Ca^2+^. However, our understanding of the role of mitochondria in regulating synaptic plasticity is incomplete. Here, we have used optical and electrophysiological techniques in cultured hippocampal neurons and *ex vivo* hippocampal slices from mice with haploinsufficiency of the mitochondrial Ca^2+^ uniporter (MCU^+/−^) to address whether reducing mitochondrial Ca^2+^ uptake alters synaptic transmission and plasticity. We found that cultured MCU^+/−^ hippocampal neurons have impaired Ca^2+^ clearance, and consequently enhanced synaptic vesicle fusion at presynapses occupied by mitochondria. Furthermore, long-term potentiation (LTP) at mossy fibre (MF) synapses, a process which is dependent on presynaptic Ca^2+^ accumulation, is enhanced in MCU^+/−^ slices. Our results reveal a previously unrecognised role for mitochondria in regulating presynaptic plasticity of a major excitatory pathway involved in learning and memory.

## Teaser

**Summary:** Optical and electrophysiological studies of MCU^+/−^ neurons reveal a previously unrecognised role for mitochondria in regulating presynaptic plasticity of a major excitatory pathway.

## INTRODUCTION

Mitochondria positioned at presynapses are an important source of ATP for powering synaptic transmission, but they also take up local Ca^2+^, thereby modulating synaptic activity (Devine and Kittler, 2018). We and others have reported that presynaptic mitochondria reduce activity-dependent local Ca^2+^ transients, reducing neurotransmission (Kwon et al., 2016; Vaccaro et al., 2017). This is mediated by Ca^2+^ uptake via the mitochondrial Ca^2+^ uniporter (MCU). MCU forms a Ca^2+^-selective pore in the inner mitochondrial membrane enabling rapid Ca^2+^ uptake into the mitochondrial matrix (Patron et al., 2013). MICU1, MICU2 and MICU3 gate Ca^2+^ entry into the MCU, thereby setting the Ca^2+^ uptake threshold (Stefani et al., 2015). MICU1 and MICU2 require high local [Ca^2+^] for Ca^2+^ uptake (Kamer and Mootha, 2015). However, in neurons, MCU is gated by the brain-specific form, MICU3, with a 10-fold lower uptake threshold (Ashrafi et al., 2020). Thus, neuronal mitochondria play a major role in Ca^2+^ homeostasis. Indeed, pharmacological MCU blockade demonstrates that presynaptic mitochondria account for ∼40% of Ca^2+^ clearance in rat sensory neurons (Shutov et al., 2013).

Presynaptic Ca^2+^ signals are important in regulating synaptic plasticity. High-frequency tetanic stimulation enhances synaptic transmission over short timescales (minutes) via post-tetanic potentiation (PTP), which is thought to be mediated by sustained elevated presynaptic Ca^2+^ due to prolonged stimulation (Katz and Miledi, 1968). Over longer timescales (hours), tetanic stimulation can be followed by a sustained increase in synaptic transmission called long-term potentiation (LTP), thought to underly learning and memory (Bliss and Lømo, 1973). LTP is governed by presynaptic mechanisms at hippocampal mossy fibre (MF) synapses, connecting dentate gyrus (DG) granule cells with CA3 neurons. MF LTP depends upon presynaptic Ca^2+^ accumulation during sustained activity (Nicoll and Schmitz, 2005). Furthermore, giant MF presynapses are replete with up to 50 mitochondria per terminal (Rollenhagen et al., 2007), and they undergo dramatic changes in synaptic strength. Therefore, this is a perfect system for studying the impact of mitochondria on short- and long-term plasticity. Pharmacological inhibition of mitochondrial Ca^2+^ uptake blocks PTP at crayfish neuromuscular junction (Tang and Zucker, 1997), and reducing MCU expression impairs short-term synaptic plasticity in cortical neurons (Kwon et al., 2016). However, whether MCU-mediated Ca^2+^ uptake modulates other forms of plasticity, or plasticity at other synapses, is unknown.

Here, we investigate how MCU shapes synaptic plasticity at hippocampal synapses in neuronal cultures and MF synapses in slices using gene-targeted MCU knockout in mouse. We show that MCU haploinsufficiency (MCU^+/−^) reduces­ mitochondrial presynaptic Ca^2+^ clearance, increasing release probability, despite reduced ATP. Strikingly, presynaptic LTP is enhanced when MCU levels are reduced. This highlights the importance of MCU-mediated presynaptic Ca^2+^ uptake in regulating basal neurotransmission and presynaptic plasticity of a major hippocampal excitatory synapse.

## RESULTS AND DISCUSSION

### MCU^+/−^ reduces presynaptic mitochondrial Ca^2+^ clearance

**Fig. 1.**
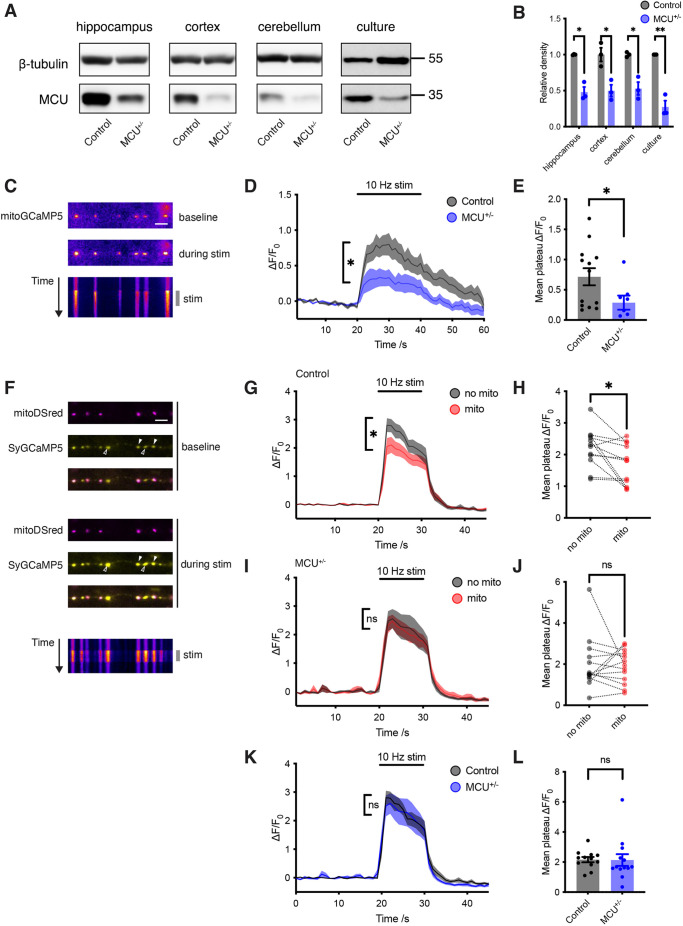
**MCU heterozygosity blocks mitochondrial presynaptic Ca^2+^ clearance.** (A) Western blot of MCU protein in lysate from 6-month-old mice (hippocampus, cortex and cerebellum), and E16 primary neuronal cultures. (B) Quantification of MCU protein level, normalised to β-tubulin (unpaired two-tailed *t*-tests, *n*=3). Hippocampus, *P=0.002; cortex, *P=0.016; cerebellum, *P=0.007; primary neuronal cultures, **P=0.001. (C) Live images of neurons transfected with mitoGCaMP5 before and during 10 Hz field stimulation (pseudocolour). Kymograph shows change in fluorescence over time (duration 60 s). Scale bar: 5 μm. (D) Average ΔF/F_0_ mitoGCaMP5 traces from control neurons (*n*=13, grey) and MCU^+/−^ neurons (*n*=8, blue) showing average increase in mitochondrial fluorescence following stimulation for 20 s (*t*=20–40) at 10 Hz. (E) Average Ca^2+^ rise in mitochondria following stimulation (average of ΔF/F_0_ measurements taken for Δ*t=*20–40 s from D). ΔF/F_0_ for mitochondria from control neurons=0.7±0.14 (*n*=13), and for mitochondria from MCU^+/−^ neurons=0.3±0.12 (*n*=8) (unpaired two-tailed *t*-test, *P=0.049). (F) Live images of neurons transfected with mitoDSred (magenta) and SyGCaMP5 (yellow) before and during 10 Hz field stimulation. Kymograph shows change in fluorescence of SyGCaMP5 over time (duration 30 s). Filled arrowheads indicate terminals occupied by mitochondria, and empty arrowheads indicate unoccupied terminals. Scale bar: 5 μm. (G) Average ΔF/F_0_ SyGCaMP5 traces from control neurons (*n*=12) plotting average of terminals without mitochondria (grey) and with mitochondria (red). Stimulation occurred for 10 s (*t*=20–40) at 10 Hz. (H) Average Ca^2+^ response following stimulation of control neurons, comparing terminals with and without a mitochondrion (average of ΔF/F_0_ measurements taken for Δ*t=*20–30 s from G). ΔF/F_0_=1.7±0.18 in terminals with mitochondria, and 2.2±0.17 in terminals without mitochondria (*n*=12) (paired two-tailed *t*-test, *P=0.016). (I) Average ΔF/F_0_ SyGCaMP5 traces from MCU^+/−^ neurons (*n*=13) plotting average of terminals without mitochondria (grey) and with mitochondria (red). Stimulation occurred for 10 s (*t*=20–40) at 10 Hz. (J) Average Ca^2+^ response following stimulation of MCU^+/−^ neurons, comparing terminals with and without mitochondria (average of ΔF/F_0_ measurements taken for Δ*t=*20–30 s from G). ΔF/F_0_=1.9±0.18 in terminals with mitochondria, and 2.0±0.18 in terminals without mitochondria (*n*=13) (paired two-tailed *t*-test; ns, not significant, *P*=0.83). (K) Average ΔF/F_0_ SyGCaMP5 traces in terminals without mitochondria, in control (*n*=12, grey) and MCU^+/−^ neurons (*n*=13, blue). Stimulation occurred for 10 s (*t*=20–40) at 10 Hz. (L) Average Ca^2+^ response following stimulation of control and MCU^+/−^ neurons, in terminals without mitochondria (average of ΔF/F_0_ measurements taken for Δ*t=*20–30 s from K). ΔF/F_0_=2.2±0.17 in terminals from control neurons (*n*=12), and 2.1±0.39 in terminals from MCU^+/−^ neurons (*n*=13) (unpaired two-tailed *t*-test; ns, not significant, *P*=0.95). Experiments were performed in E16 mouse hippocampal neuronal cultures at 10–12 DIV. Error bars represent s.e.m.

We used MCU^+/−^ animals because MCU KO is embryonic lethal on the C57BL/6 background (Murphy et al., 2014). We confirmed that MCU protein levels are reduced in MCU^+/−^ animals compared to wild-type (WT) littermate controls, throughout the brain and in neuronal cultures (Fig. 1A,B). In MCU^+/−^ animals, MICU3 was reduced in all brain areas examined and MICU1 was reduced in hippocampus and cortex, compared to controls (Fig. S1A,B). There were no gross anatomical differences between MCU^+/−^ and control neurons (Fig. S2A–E).

We then live-imaged neurons with a mitochondrially targeted Ca^2+^ reporter (mitoGCaMP5) (Fig. 1C). Mitochondrial Ca^2+^ uptake in response to neuronal stimulation was significantly lower in MCU^+/−^ neurons compared to controls (Fig. 1D,E). We imaged presynaptic Ca^2+^ transients in response to stimulation using a presynaptically targeted Ca^2+^ reporter (SyGCaMP5) (Fig. 1F). By co-transfecting neurons with a fluorescent mitochondrial reporter (MtDsRed), we compared Ca^2+^ transients at boutons occupied by mitochondria with those at unoccupied boutons. We confirmed that, in control neurons, boutons occupied by mitochondria exhibit lower Ca^2+^ transients than unoccupied boutons (Fig. 1G,H) (Vaccaro et al., 2017). In contrast, Ca^2+^ transients at boutons in MCU^+/−^ neurons were not altered by mitochondria (Fig. 1I,J). Ca^2+^ transients in boutons without mitochondria were identical in MCU^+/−^ and controls, confirming that altering MCU gene dosage specifically impacts mitochondrial Ca^2+^ uptake (Fig. 1K,L).

Overall, MCU^+/−^ reduces mitochondrial Ca^2+^ uptake sufficiently to block the effect of mitochondria on presynaptic Ca^2+^.

### MCU^+/−^ enhances synaptic vesicle fusion and alters activity-dependent redistribution of mitochondria

**Fig. 2.**
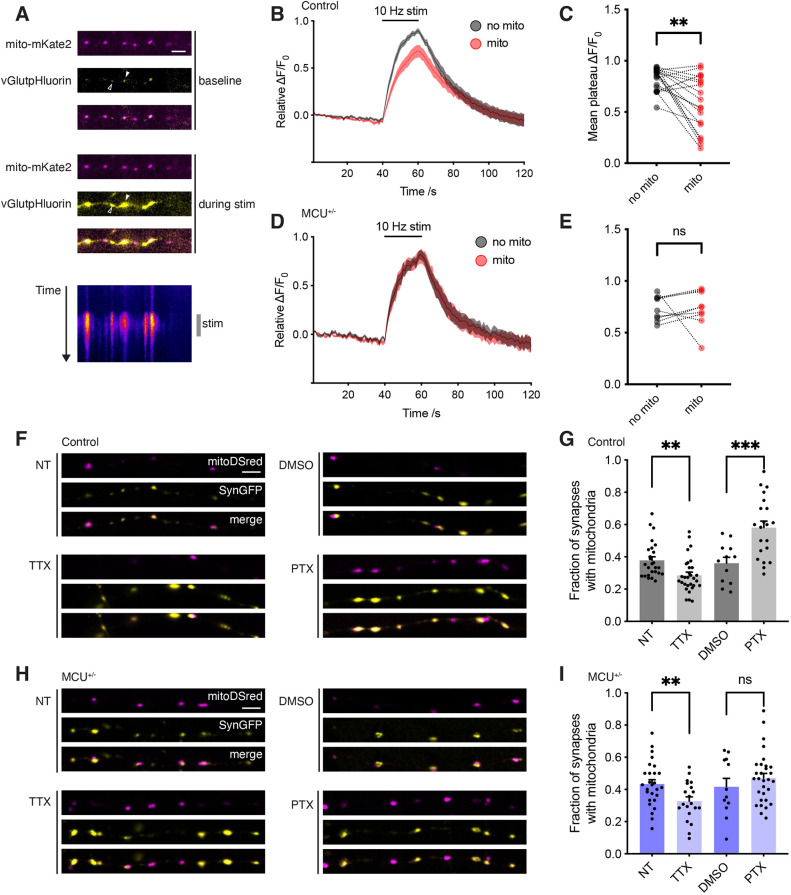
**Functional impact of MCU heterozygosity on SV fusion and activity-dependent redistribution of mitochondria.** (A) Live images of neurons transfected with mito–mKate2 (magenta) and vGlutpHluorin (yellow) before and during 10 Hz field stimulation. Kymograph shows change in fluorescence of vGlutpHluorin over time (duration 70 s). Filled arrowhead indicates synaptic terminal occupied by mitochondria, and empty arrowhead indicates an unoccupied terminal. (B) Average ΔF/F_0_ vGlutpHluorin traces from control neurons (*n*=19) plotting average of terminals without mitochondria (grey trace) and with mitochondria (red trace). Stimulation occurred for 20 s (*t*=40–60) at 10 Hz. (C) Average mean vGlutpHluorin response following stimulation of control neurons (average of ΔF/F_0_ measurements taken for Δ*t=*50–60 s from B). ΔF/F_0_=0.59±0.06 in terminals with mitochondria, and 0.82±0.03 in terminals without mitochondria (*n*=19) (paired two-tailed *t*-test, **P=0.003). (D) Average ΔF/F_0_ vGlutpHluorin traces from MCU^+/−^ neurons (*n*=9) plotting average of terminals without mitochondria (grey) and with mitochondria (red). Stimulation occurred for 20 s (*t*=40–60) at 10 Hz. (E) Average mean vGlutpHluorin response following stimulation of MCU^+/−^ neurons (average of ΔF/F_0_ measurements taken for Δ*t=*50–60 s from D). ΔF/F_0_=0.73±0.04 in terminals with mitochondria, and 0.73±0.06 in terminals without mitochondria (*n*=9) (paired two-tailed *t*-test; ns, not significant, *P*=0.99). (F) Confocal images of fixed control neurons transfected with mitoDSred (magenta) and synaptophysin–GFP (SynGFP; yellow). Neurons are either non-treated (NT), TTX-treated (1 μM, 48 h), DMSO-treated (1:2000, 48 h) or PTX-treated (100 μM, 48 h). Scale bar: 5 μm. g, Fraction of SynGFP clusters colocalising with mitochondria in control neurons. NT 0.38±0.02 (*n*=26), TTX 0.29±0.02 (*n*=30) (unpaired two-tailed *t*-test, **P=0.002). DMSO 0.36±0.04 (*n*=12), PTX 0.58±0.04 (*n*=21) (unpaired two-tailed *t*-test, ***P=0.001). (H) Confocal images of neuronal processes of fixed MCU^+/−^ neurons transfected with mitoDSred (magenta) and SynGFP (yellow), with the same conditions as F. (I) Fraction of SynGFP clusters co-localising with mitochondria in MCU^+/−^ neurons. NT 0.43±0.03 (*n*=27), TTX 0.33±0.03 (*n*=20) (unpaired two-tailed *t*-test; ns, not significant, *P=*0.35). DMSO 0.42±0.05 (*n*=12), PTX 0.47±0.03 (*n*=30) (unpaired two-tailed *t*-test, **P=0.008). Scale bars: 5 μm. Experiments were performed in E16 mouse hippocampal neuronal cultures at 10–12 DIV. Error bars represent s.e.m.

To image synaptic vesicle (SV) fusion we used vGlut1pHluorin, while simultaneously imaging mitochondria with mitochondrially targeted LSS-mKate2 (Fig. 2A). In controls, SV fusion was reduced at boutons occupied by mitochondria (Fig. 2B,C). In contrast, presynaptic mitochondria in MCU^+/−^ neurons failed to alter SV fusion (Fig. 2D,E). Therefore, MCU^+/−^ blocks mitochondria from reducing Ca^2+^ clearance and consequent SV fusion at presynapses. MCU knockdown reportedly accelerates SV endocytosis by reducing mitochondrial Ca^2+^ efflux following neuronal stimulation, thereby lifting the breaking effect of Ca^2+^ on SV endocytosis (Marland et al., 2016). In agreement, we found accelerated SV endocytosis in MCU^+/−^ neurons compared to controls (Fig. S3).

To exclude the possibility that MCU^+/−^ enhances SV fusion by enhancing ATP availability, we used PercevalHR (Tantama et al., 2013) to compare ATP levels in MCU^+/−^ neurons and controls following neuronal stimulation. ATP was lower in MCU^+/−^ (Fig. S4A–D) consistent with the requirement for mitochondrial Ca^2+^ uptake to drive ATP production (Ashrafi et al., 2020).

Mitochondria redistribute to and from presynapses in response to prolonged (48 h) alterations in network activity, contributing to homeostatic plasticity (Vaccaro et al., 2017). We examined the impact of MCU^+/−^ on this redistribution by co-transfecting neurons with synaptophysin–GFP to label presynapses, and mitoDsRed to label mitochondria. We determined presynaptic mitochondrial occupancy in control and MCU^+/−^ neurons at baseline, and following 48 h treatment with tetrodotoxin (decreasing network activity) or picrotoxin (increasing network activity). In control (Fig. 2F,G) and MCU^+/−^ neurons (Fig. 2H,I), lowering activity reduced presynaptic mitochondrial occupancy. However, increasing activity failed to recruit mitochondria to presynapses in MCU^+/−^ neurons, in contrast to controls (Fig. 2I). Following picrotoxin treatment, presynaptic mitochondrial occupancy in MCU^+/−^ neurons was significantly lower than in controls (two-tailed unpaired *t*-test, *P*=0.0271), suggesting that MCU-mediated Ca^2+^ uptake is required for mitochondrial recruitment to presynapses. This is consistent with previous work demonstrating that increasing mitochondrial matrix Ca^2+^ ceases mitochondrial movement (Chang et al., 2011).

### Increased MF excitability and enhanced presynaptic LTP in MCU^+/−^ mice

**Fig. 3.**
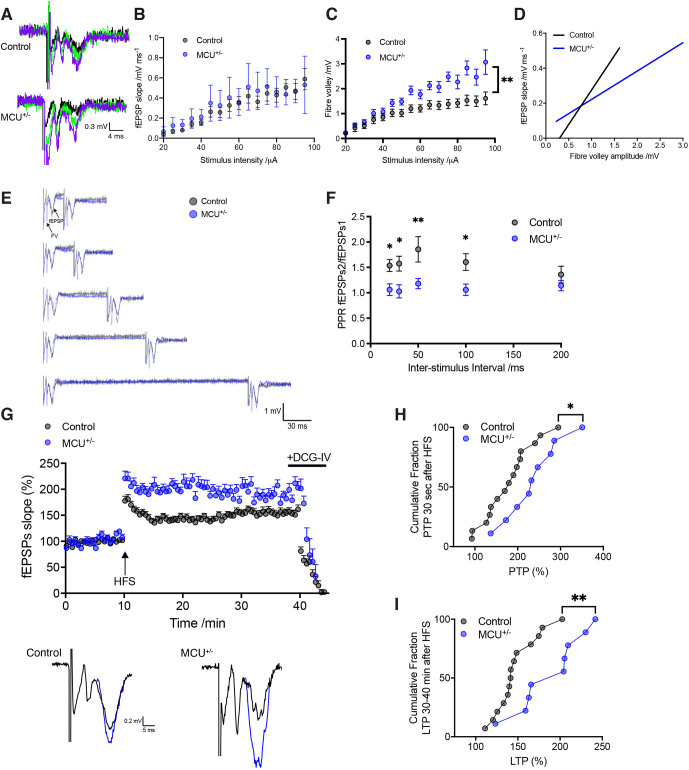
**Larger fibre volley amplitude, loss of paired-pulse facilitation and increased PTP and LTP at the DG–CA3 MF synapse in MCU^+/−^ hippocampal slices.** (A) Representative traces of DG–CA3 MF fEPSPs are shown for control (top) and MCU^+/−^ (bottom). The responses to a range of different stimulus intensities (30–80 µA) are superimposed. (B) Input–output relationship for control (black circles) and MCU^+/−^ (blue circles) showing no difference in basal transmission (control *n=*13 slices; MCU^+/−^ *n=*10 slices; two-way repeated measures ANOVA with Bonferonni correction, *P=*0.45). (C) Input–output relationship for fibre volleys showing higher fibre volley amplitudes in MCU^+/−^ (control *n=*13 slices; MCU^+/−^ *n=*10 slices; two-way repeated measures ANOVA with Bonferonni correction, **P=0.01). (D) MF fEPSP slope plotted against fibre volley amplitude for the same data (linear regression analysis, two-way repeated measures ANOVA with Bonferonni correction, ****P<0.0001). (E) Examples of PPR in control neurons (grey) and MCU^+/−^ (blue) for a range of inter-stimulus intervals. (F) Mean PPR was reduced at inter-stimulus intervals of between 20 and 100 ms in the MCU^+/−^ mouse (control *n=*12 slices; MCU^+/−^ *n=*12 slices). Unpaired non-parametric Mann–Whitney test: 20 ms *P=0.0188, 30 ms *P=0.0112, 50 ms **P=0.0015, 100 ms *P=0.03, 200 ms *P*=0.4562. (G) Normalised fEPSP slope measurements for control (black circles) and MCU^+/−^ (blue circles) before and after LTP induction (arrow). Representative traces of fEPSPs before (black) and after LTP induction (blue) from control (left) and MCU^+/−^ (right) shown below. (H) Cumulative fraction of PTP showing a shift towards higher values in MCU^+/−^ slices for the first 3 min after LTP induction (control 178.5±14.88%, MCU^+/−^ 236.3±21.32%, *n*=9–15, unpaired two-tailed *t*-test, *P=0.0323). (I) Cumulative fraction of LTP 30–40 min after HFS train showing a shift towards higher LTP% in MCU^+/−^ slices (control 147.6±6.65%, MCU^+/−^ 189.2±12.83%, *n*=9–14, unpaired two-tailed *t*-test, **P=0.0048). Experiments were performed in acute hippocampal slices from mice at 1.5–2 months of age. Error bars represent s.e.m.

Next, given that hippocampal MF boutons are replete with mitochondria, and that plasticity at these synapses is expressed presynaptically, we asked whether MCU^+/−^ changes synaptic plasticity at MF synapses. We examined basal synaptic transmission by plotting the slope (output) of evoked field excitatory postsynaptic potential (fEPSP) in response to increasing stimulation intensity (input). Input–output curves from MCU^+/−^ slices were similar to WT littermate controls (Fig. 3A,B). We observed higher fibre volley amplitudes in MCU^+/−^ at all but the weakest stimulus strengths (Fig. 3C), suggesting increased presynaptic strength. We then compared fEPSP slope against fibre volley amplitude. MCU^+/−^ exhibited higher amplitude fibre volleys for equivalent fEPSP slope values compared to controls (Fig. 3D). These results suggest that DG–CA3 transmission is increased in MCU^+/−^.

Paired-pulse facilitation (a form of presynaptic short-term plasticity) is large at MF synapses (Salin et al., 1996). It is assessed by induction of two closely spaced stimuli. The characteristic paired-pulse facilitation of DG-evoked EPSPs in control slices was absent in MCU^+/−^, at intervals less than 200 ms (Fig. 3F). As the paired-pulse ratio (PPR) inversely correlates with release probability, and is attributed to presynaptic Ca^2+^, this result is consistent with enhanced release probability in MCU^+/−^ at shorter intervals (20–100 ms).

Finally, we examined the role of MCU^+/−^ on LTP elicited by long high-frequency stimulation delivered to stratum granulosum. PTP [resulting from slow efflux of accumulated mitochondrial Ca^2+^ (Lee et al., 2007; Tang and Zucker, 1997)], was increased in MCU^+/−^ compared to controls (Fig. 3G,H). Moreover, LTP in MCU^+/−^ was greater than in controls (Fig. 3G,I). DCG-IV (1 μM) had a depressant effect, consistent with the high sensitivity of MFs to group II metabotropic glutamate receptor agonists, confirming that measured LTP was specific to MF synapses.

Our results show that presynaptic mitochondrial Ca^2+^ uptake via MCU limits both SV fusion and endocytosis, and LTP, despite increasing ATP. We predict that the observed increase in MF LTP in MCU^+/−^ would correlate with enhanced learning and memory. Although a different form of LTP, increasing hippocampal N-methyl-D-aspartate (NMDA) receptor activity enhances neurotransmission and LTP at Schaffer-CA1 synapses, with improved learning and memory in mice (Tang et al., 1999). Furthermore, pharmacological inhibition of DG–CA3 transmission impairs spatial learning in mice (Lassalle et al., 2000).

Mitochondria sense changes in cytoplasmic Ca^2+^ and energy usage, which are both indicators of neuronal activity, and presynaptic mitochondria can alter synaptic activity via MCU-mediated Ca^2+^ uptake (Kwon et al., 2016; Vaccaro et al., 2017). This ability to both detect and change neuronal activity suggests that mitochondria could regulate neuronal network activity (Ruggiero et al., 2021). Mitochondria have been shown to regulate network activity of hippocampal neurons, specifically the mean firing rate set point (Styr et al., 2019). Our data suggest that mitochondria also regulate the set point of MF LTP.

That MCU sets MF LTP at a submaximal level might help protect MF synapses from excitotoxicity. By analogy, NMDA receptor levels are downregulated at postsynapses in the adult brain (Bai et al., 2004; Law et al., 2003), which potentially protect Schaffer–CA1 synapses from excitotoxicity by limiting Ca^2+^ flux through NMDA receptors. However, aged mice with NMDA receptor overexpression maintain enhanced learning and memory performance (Cao et al., 2007), suggesting that long-term enhancement of LTP is not necessarily deleterious. Mitochondria might reduce the LTP set point to conserve resources, because enhanced neurotransmission and LTP in MCU^+/−^ mice is likely metabolically expensive. Mitochondria can meet the energy requirements of active synapses through ATP provision (Pathak et al., 2015; Rangaraju et al., 2014). Moreover, when glucose is limited, neuronal metabolism switches to oxidative phosphorylation using lactate or pyruvate as fuel, and active synapses need MCU-mediated Ca^2+^ uptake to drive ATP production under these conditions (Ashrafi et al., 2020). Therefore, in real-world conditions of constrained food – and therefore fuel – availability, mitochondria that avidly take up Ca^2+^ via MCU are important for maintaining ATP delivery. Constrained neurotransmission and LTP might be a consequence of enabling this metabolic flexibility. The need to balance accurate neurotransmission with fuel availability is highlighted by recent work showing that the neocortex saves energy during food scarcity by sacrificing coding precision (Padamsey et al., 2021).

In summary, presynaptic mitochondria are able to couple synaptic activity to fuel availability, and therefore limit presynaptic Ca^2+^ at active synapses. This could potentially avert catastrophic energy shortages in active synapses, and neuronal toxicity due to Ca^2+^ overload.

## MATERIALS AND METHODS

### Animal models

The mouse line Mcu^tm1b(EUCOMM)Hmgu^ was obtained from MRC Harwell, UK. MCU homozygous knockout animals are non-viable on this genetic background (C57BL/6), but heterozygous animals are viable and can breed. In contrast, MCU homozygous knockout animals on the outbred CD1 background are viable (Pan et al., 2013). Animals were maintained under controlled conditions (temperature 20±2°C; 12 h light–12 h dark cycle). Food and water were provided *ad libitum*. All experimental procedures were carried out in accordance with institutional animal welfare guidelines and licensed by the UK Home Office in accordance with the Animals (Scientific Procedures) Act 1986. All data involving procedures carried out in animals are reported in compliance with ARRIVE guidelines (Kilkenny et al., 2010).

### Neuronal cultures and transfection

Primary hippocampal cultures were prepared as previously described from embryonic day (E)16 mice of either gender (Vaccaro et al., 2017). Following 15 min treatment with 0.25% trypsin and trituration, cells were plated on poly-L-lysine-coated round 12 mm coverslips for fixed experiments or 25 mm coverslips for live experiments at a density of 250,000 per 3 cm well. Neurons were transfected by lipofection with Lipofectamine 2000 at 7 days *in vitro* (DIV) and then imaged at 10–12 DIV.

### Western blotting

SDS-PAGE and western blotting samples were denatured at 94°C for 5 min in 3× SDS sample buffer (150 mM Tris-HCl pH 8, 6% SDS, 0.3 M DTT, 0.3% Bromophenol Blue and 30% glycerol). Polyacrylamide gels were prepared using 10% running gels and 5% stacking gels in Novex 1.5-mm cassettes and run using the Novex XCell SureLock Mini-Cell system. Gels were transferred onto Hybond-C nitrocellulose membrane (GE Healthcare). Membranes were blocked in 4% milk powder in phosphate-buffered saline with 0.05% Tween 20 (PBS-T) for 1–2 h. Antibodies were incubated in blocking solution (overnight at 4°C for primary antibodies or 1 h at RT for HRP-conjugated secondary antibodies). Membranes were developed using the ECL-Plus reagent (GE Healthcare) and acquired in a chemiluminescence imager coupled to a CCD camera (ImageQuant LAS 4000 mini). Densitometric analysis was performed using ImageJ software (https://imagej.nih.gov/ij/). Original blot images are shown in Figs S5 and S6.

### Antibodies, DNA constructs and reagents

SyGCaMP5, mitoDsRed, synaptophysin–GFP, vGlut1pHluorin and Mito-LSSmKate2 have been previously described (Vaccaro et al., 2017). MitoGCaMP5 was obtained from Addgene (#58509; VV073: 1xCox8 -GCaMP5G in fubi). GW1-PercevalHR was Addgene plasmid #49082, deposited by Gary Yellen. MCU antibody was purchased from Atlas Antibodies (HPA016480) and used at 1:1000. Other antibodies used were: Anti-β-tubulin (Sigma T8328) at 1:2000, anti-MCUb (Abcepta AP12355b) at 1:500, anti-MCUR1 (Aviva ARP44777_P050) at 1:500, anti-MICU1 (Atlas HPA037480) at 1:500, anti-MICU2 (Abcam AB101465) at 1:500, anti-MICU3 (Atlas HPA024771) at 1:500, anti-NCLX (Abcam AB83551) at 1:500, anti-LETM1 (Atlas HPA011029) at 1:500, anti-VDAC1 (NeuroMab 75-204) at 1:1000. Picrotoxin (PTX) was purchased from Sigma-Aldrich and used at 100 μM; tetrodotoxin (TTX) was purchased from Tocris Bioscience and used at 1 μM.

### Fixed imaging

After fixation using 4% PFA for 5 min, cells were washed twice and blocked in PBS solution containing 10% horse serum, 0.5% BSA and 0.2% Triton X-100. Images of fixed cultures were taken on a Zeiss LSM700 confocal using a 63× oil objective (NA 1.4) and a 20× water objective (NA 1.0).

### Live imaging

For measurement of Ca^2+^ and synaptic vesicle fusion, experiments were performed at 37°C while perfusing the coverslips in external solution containing 125 mM NaCl, 10 mM D-glucose, 10 mM HEPES, 5 mM KCl, 2 mM CaCl_2_ and 1 mM MgCl_2_, which was brought to a pH of 7.4 using NaOH. An inverted Zeiss Axiovert 200 microscope and a 63× oil objective (NA 1.4) coupled to a Photometrics Evolve camera were used to image frames with 30 ms exposure at 1 frame per second in the software Micro-Manager (Edelstein et al., 2010). Using Chroma filters, coverslips were excited through a D470/40X filter and emission was split using an Optosplit II (Cairn Research) (Awabdh et al., 2016) and a 565DCXR dichroic thereby collecting with HQ522/40 M and HQ607/75 M filters for SyGCaMP5 or VGlut1pHluorin and MtDsRed or Mito-LSSmKate2, respectively. Presynapses with and without mitochondria were discriminated on the basis of colocalisation of SyGCaMP5 or vGlut1pHluorin and MtDsRed or Mito-LSSmKate2, respectively – signal overlap for the duration of the imaging period was required for a synapse to be deemed occupied by a mitochondrion. Clear separation in signal was required to deem a synapse devoid of mitochondria. Field stimulation was achieved using a Grass S9 or S88 stimulator and a Warner Instruments stimulation bath. Individual stimulating pulses lasted for 1 ms and were set at 10 V as part of stimulation trains at 10 Hz, with durations of 10 s or 20 s.

For ATP measurements, primary hippocampal neurons were transfected with the ratiometric ATP:ADP sensor PercevalHR (Tantama et al., 2013) at 10 DIV using Lipofectamine 2000. After 72 h, imaging of neurons was performed using a Zeiss LSM700 confocal microscope with a 63× water immersion objective. PercevalHR was excited at 405 nm and at 488 nm, and emission was collected at wavelengths longer than 510 nm. Images were acquired every 2 s for 200 s. Neurons were stimulated using 20 μM glutamate for 10 s at 30 s. ATP:ADP ratios over time were obtained in ImageJ by taking regions of interest (ROIs) in each neuron, measuring average fluorescence in the ROIs for each channel and calculating the 488 nm/405 nm ratio. Mitochondrial Ca^2+^ uptake in response to glutamate stimulation was also measured by imaging MitoGCaMP5 using the confocal microscope, to verify that both glutamate stimulation and field stimulation produced an equivalent mitochondrial response. MitoGCaMP5G was excited at 488 nm and emission was collected at wavelengths longer than 510 nm. Images were acquired every 2 s for 200 s and neurons were stimulated using 20 μM glutamate for 10 s at 30 s. Mito-GCaMP5G fluorescence over time was obtained in ImageJ by taking ROIs in each neuron, measuring average fluorescence in the ROIs.

### Field EPSP recordings

Transverse hippocampal slices (300 μm) were prepared from 5–8-week-old mice (MCU^+/−^ males only, littermates) and obtained using a vibratome (Leica, VT-1200S). Brain was removed and kept in ice-cold dissecting solution. Slices were stored at 35°C for 30 min after slicing and then at 22°C. For the dissection and storage of slices, the solution contained (in mM): 87 NaCl, 25 NaHCO_3_, 10 glucose, 75 sucrose, 2.5 KCl, 1.25 NaH_2_PO_4_, 0.5 CaCl_2_ and 7 MgCl_2_, saturated with 95% O_2_/5% CO_2_.

During experiments, slices were superfused with artificial cerebrospinal fluid solution (ACSF) containing: NaCl (125 mM), NaHCO_3_ (25 mM), glucose (25 mM), KCl (2.5 mM), NaH_2_PO_4_ (1.25 mM), CaCl_2_ (2 mM) and MgCl_2_ (2 mM), equilibrated with 95% O_2_/5% CO_2_. The osmolarity and pH of perfusion solutions were adjusted to ∼320 mOsmol/l and 7.3, respectively. All recordings were performed at room temperature (21–22°C). A slice was transferred into the recording chamber and visualised with an Olympus BX 51WI microscope (Olympus Europa Holding GmbH, Hamburg, Germany) connected to a KPM-3 Hitachi infrared video camera. A bipolar stimulating electrode (FHC Inc., Bowdoin, Maine, USA) was positioned under low magnification (10×) in the supra-granular blade of the DG to activate MF synapses every 10 s using constant current (0.2–1 μA, 80 μs square pulses). A field recording electrode was placed in stratum lucidum in CA3. To set the intensity of stimulation, an input-output (I/O) relationship was obtained for each slice when applying the control perfusion solution. The stimulus intensity was set such that the amplitude of the test fEPSP reached around 40% of maximum amplitude based on the I/O curve. Synaptic responses were recorded with an Axopatch 200B amplifier (Molecular Devices), filtered at 2 kHz (internal 4-pole low-pass Bessel filter), and sampled at 10 kHz. Two tests were routinely applied to verify that the signal recorded in stratum lucidum was a MF fEPSP. First, increasing the stimulation frequency caused pronounced facilitation (>2.5-fold at 1 Hz at room temperature) (Salin et al., 1996). Second, application of the group II metabotropic glutamate receptor (mGluR) agonist DCG-IV (1 μM; Tocris Bioscience) depressed the fEPSP amplitude to <20% of control fEPSP (Kamiya et al., 1996). MF LTP was induced by long high-frequency tetanic stimulation (l-HFS) represented by 100 pulses in 1 s, 3 times, separated by 10 s. All LTP recordings were performed with the γ-aminobutyric acid type A (GABA_A_) receptor antagonist bicuculline methiodide (10 μM; Abcam), and NMDA receptor antagonist *D*-APV (25 μM; Abcam) added to the ACSF to exclude any contribution from NMDA-dependent LTP.

### Data analysis

Movies were aligned using the Cairn Image Splitter plugin in ImageJ. Graphs showing ΔF/F_0_ were plotted using Mathematica software (Wolfram Research). ROIs were manually drawn and after background subtraction, fluorescence was normalised to the first 10 frames. Mean stimulation fluorescence was calculated as an average across a plateau equating to stimulation duration. For SV endocytosis, non-linear regression was used to fit a monoexponential function to the post-stimulation vGlutpHluorin data to derive the time constant (τ) of endocytosis. Exponentials were fitted between 1–60 s after termination of stimulation.

For colocalisation analysis, a region measuring 40×40 μm was chosen at least 300 μm from the soma. Colocalisation of synaptophysin–GFP and MtDsRed was quantified as the fraction of synaptophysin–GFP clusters that overlapped with at least one MtDsRed-positive pixel. Images were thresholded in ImageJ and, using the Image Calculator tool, a third image was generated showing those pixels which were positive in both input channels. Using the Particle Analysis tool, the size and number of the thresholded clusters was analysed. Microsoft Excel was used to calculate the fraction of MtDsRed-positive synaptophysin–GFP clusters. GraphPad Prism was used to perform *t*-tests and to visualise bar charts.

For each LTP recording, fEPSP amplitude after LTP induction were normalised to the amplitude measured during 10 min recorded baseline. The overall change in amplitude for *n* recordings from individual slices (from at least three animals) was determined by averaging normalised amplitudes, then expressed as a percentage.

### Statistics

For optical data, a paired two-tailed *t*-test was used to calculate statistical significance, whereby terminals with and without mitochondria within the same axon were compared. Unpaired two-tailed *t*-tests were used to compare neurons from control and MCU^+/−^ animals. Error bars represent s.e.m. Extra sum-of-squares *F* test was used to compare time constant (τ) of SV endocytosis between control and MCU^+/−^ neurons. For each LTP recording, fEPSP amplitude was normalised to the amplitude measured 10–15 min before HFS. The overall change in amplitude for *n* recorded neurons was determined by averaging normalised amplitudes, and is expressed as a percentage. For paired-pulse ratio (PPR) measurements, the average of five responses was taken for each interval for each slice. PPR was determined by dividing the peak amplitude of fEPSP2 by that of fEPSP1 using variable inter-stimulus intervals (20–200 ms). Unless otherwise noted, we routinely applied an unpaired two-tailed *t*-test to test the difference between the sampled means (Gaussian data scatter). Data were considered significant if *P*<0.05. Values are given as mean±s.e.m. Error bars represent s.e.m.

## Supplementary Material

10.1242/joces.259823_sup1 Supplementary information
