# Supplementary material for: Mitochondrial Ca2+ uniporter haploinsufficiency enhances long-term potentiation at hippocampal mossy fibre synapses
Source: J Cell Sci. 2022 Nov 23;135(22):jcs259823. doi: 10.1242/jcs.259823 (PMC10563808; doi:10.1242/jcs.259823)
Supplement: Supplementary information [file joces-135-259823-s1.pdf]

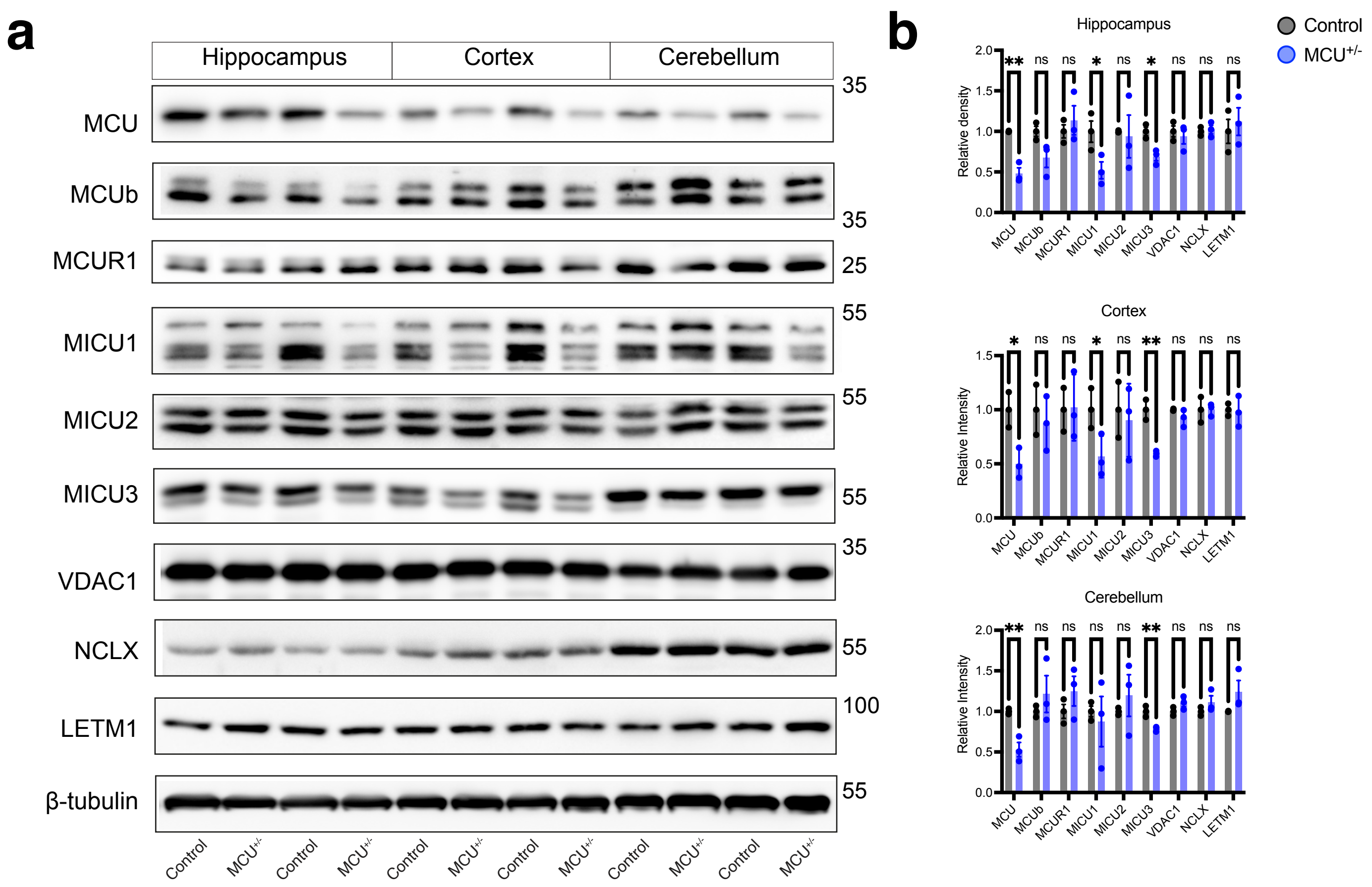

Fig. S1. Mitochondrial Ca^{2+} uptake protein levels in $MCU^{+/-}$ animals.

a, Western blot for mitochondrial Ca^{2+} uptake proteins in lysate from 6 month old mice (hippocampus, cortex and cerebellum).

b, Quantification of protein level, normalised to β-tubulin.

Hippocampus, $n = 3$, unpaired *t*-tests: MCUb $P = 0.07$, MCUR1 $P = 0.53$, MICU1 $^*P = 0.046$, MICU2 $P = 0.83$, MICU3 $^*P = 0.01$, VDAC1 $P = 0.64$, NCLX $P = 0.74$, LETM1 $P = 0.62$.

Cortex, $n = 3$, unpaired *t*-tests: MCUb $P = 0.56$, MCUR1 $P = 0.93$, MICU1 $^*P = 0.044$, MICU2 $P = 0.71$, MICU3 $^{**}P = 0.001$, VDAC1 $P = 0.16$, NCLX $P = 0.97$, LETM1 $P = 0.85$.

Cerebellum, $n = 3$, unpaired *t*-tests: MCUb $P = 0.40$, MCUR1 $P = 0.28$, MICU1 $P = 0.71$, MICU2 $P = 0.49$, MICU3 $^{**}P = 0.007$, VDAC1 $P = 0.13$, NCLX $P = 0.25$, LETM1 $P = 0.16$.

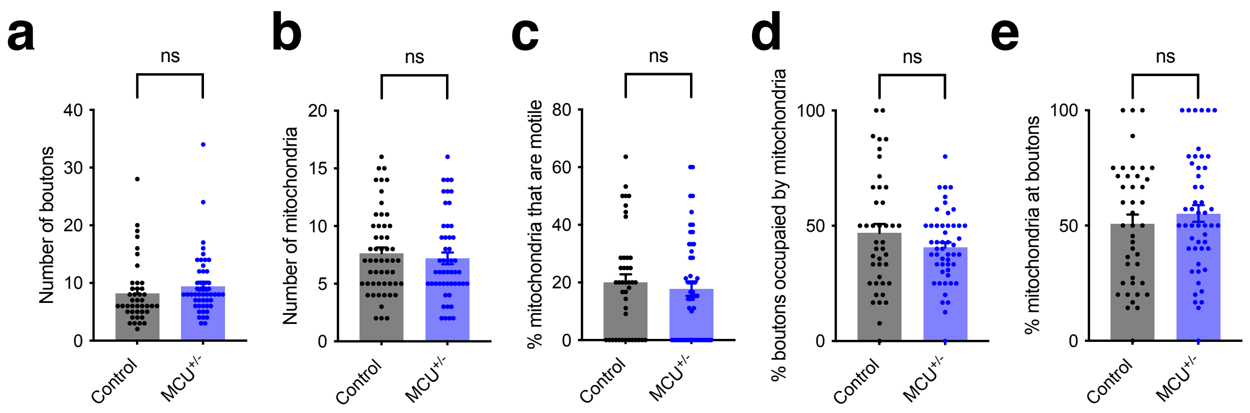

Fig. S2. Characterisation of individual neurons from $MCU^{+/-}$ and control cultures.

a, Number of boutons in control and $MCU^{+/-}$ neurons: 8.2 ± 0.83 and 9.5 ± 0.76 respectively, n = 42-50 neurons, unpaired t-test, P = 0.26.

b, Number of mitochondria in control and $MCU^{+/-}$ neurons: 7.6 ± 0.50 and 7.2 ± 0.51 respectively, n = 42-50 neurons, unpaired t-test, P = 0.54.

c, Percentage of motile mitochondria in control and $MCU^{+/-}$ neurons: 20 ± 2.8 and 18 ± 2.5 respectively, n = 42-50 neurons, unpaired t-test, P = 0.56.

d, Percentage of boutons occupied by mitochondria in control and $MCU^{+/-}$ neurons: 47 ± 3.9 and 41 ± 2.2 respectively, n = 42-50 neurons, unpaired t-test, P = 0.39.

e, Percentage of mitochondria at boutons in control and $MCU^{+/-}$ neurons: 51 ± 4.0 and 55 ± 3.6 respectively, n = 42-50 neurons, unpaired t-test, P = 0.41.

Experiments were performed in E16 mouse hippocampal neuronal cultures at DIV 10-12. Error bars represent SEM.

Journal of Cell Science • Supplementary information

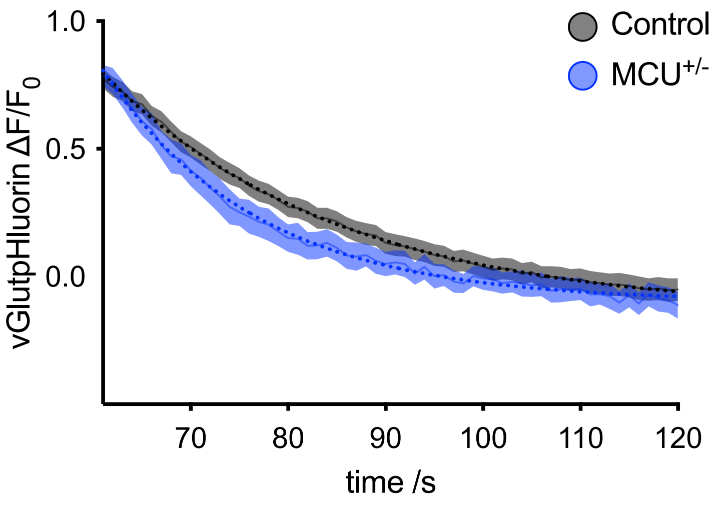

Fig. S3. MCU heterozygosity accelerates synaptic vesicle endocytosis.

Average vGlutpHluorin τ shown by dotted lines for control (grey) and MCU$^{+/-}$ neurons (blue). MCU$^{+/-}$ τ = 15.72s (95% CI 13.6 to 18.39), n = 18 neurons; Control τ = 23.95s (95% CI 20.72 to 28.18), n = 36 neurons). F statistic 13.11 (***P = 0.0003).

Journal of Cell Science • Supplementary information

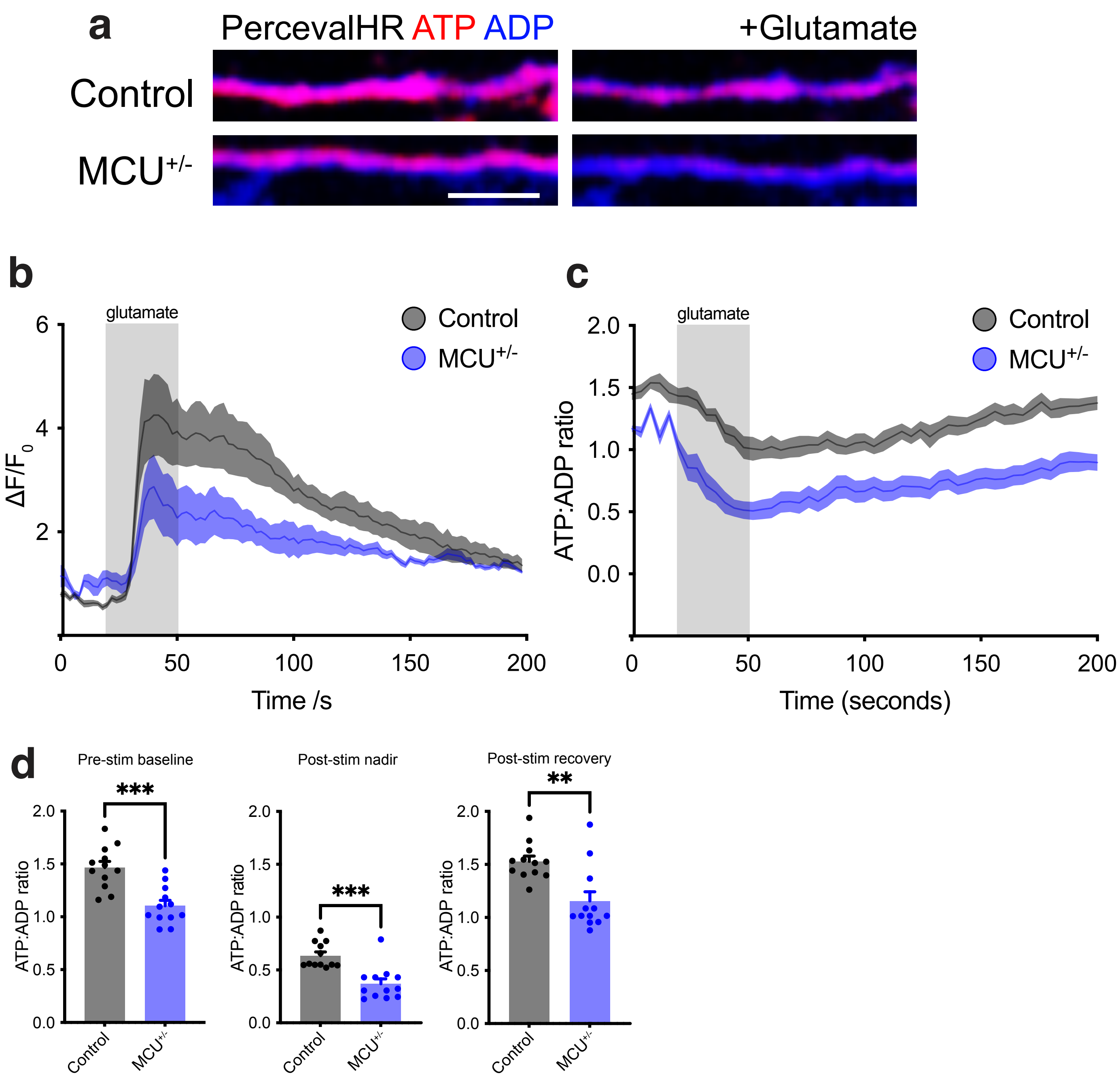

Fig. S4. ATP availability is reduced in MCU$^{+/-}$ neurons.

a, Live images of neurons transfected with PercevalHR before and during glutamate stimulation, shown in false colour. Scale bar, 10 µm.

b, Average $\Delta F/F_0$ MitoGCaMP5 traces from control neurons (n = 12, grey) and MCU$^{+/-}$ neurons (n = 12, blue). Stimulation with glutamate occurred for 30 s (t = 20—50).

c, Average ATP:ADP ratio (derived from PercevalHR imaging) from control neurons (n = 12, grey) and MCU$^{+/-}$ neurons (n = 12, blue). Stimulation with glutamate occurred for 30 s (t = 20—50).

d, Average ATP:ADP ratio (derived from PercevalHR imaging): at baseline prior to glutamate stimulation (Pre-stim baseline), the nadir following glutamate stimulation (Post-stim nadir), and at the end of the imaging period following glutamate stimulation (Post-stim recovery). ATP:ADP ratio was lower in MCU$^{+/-}$ neurons at all three time points. Pre-stim baseline, unpaired t-test, ***P = 0.0001. Post-stim nadir, unpaired t-test, ***P = 0.0001. Post-stim recovery unpaired t-test, **P = 0.0013.

Journal of Cell Science • Supplementary information

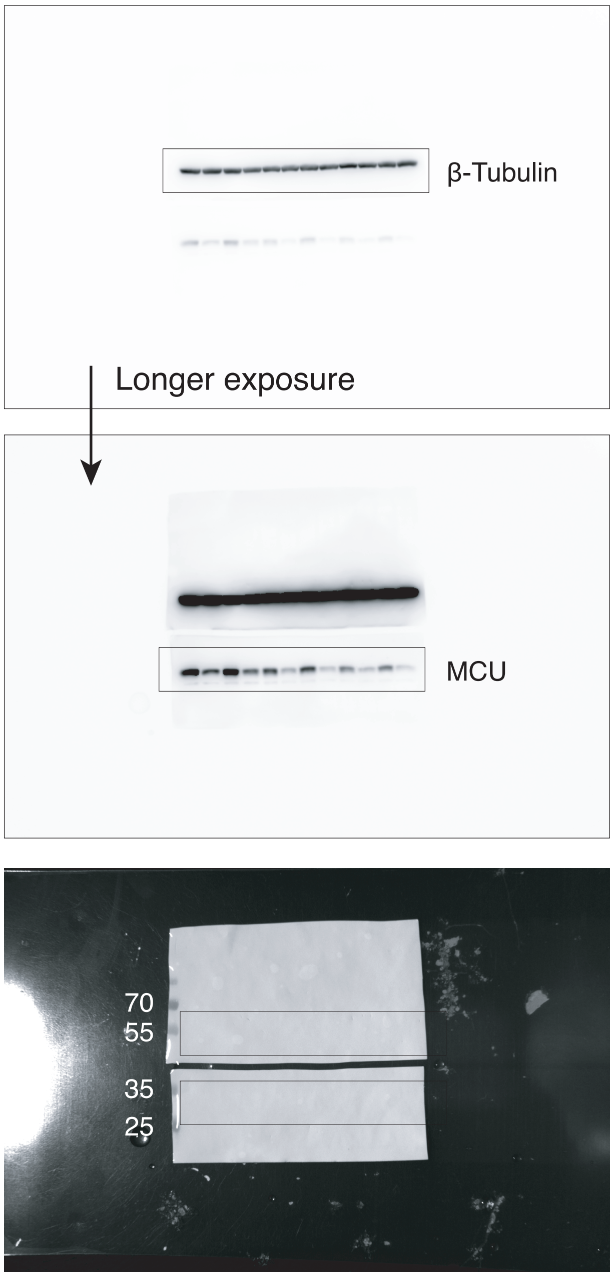

Fig. S5. Blot Transparency related to Figure 1.

Journal of Cell Science • Supplementary information

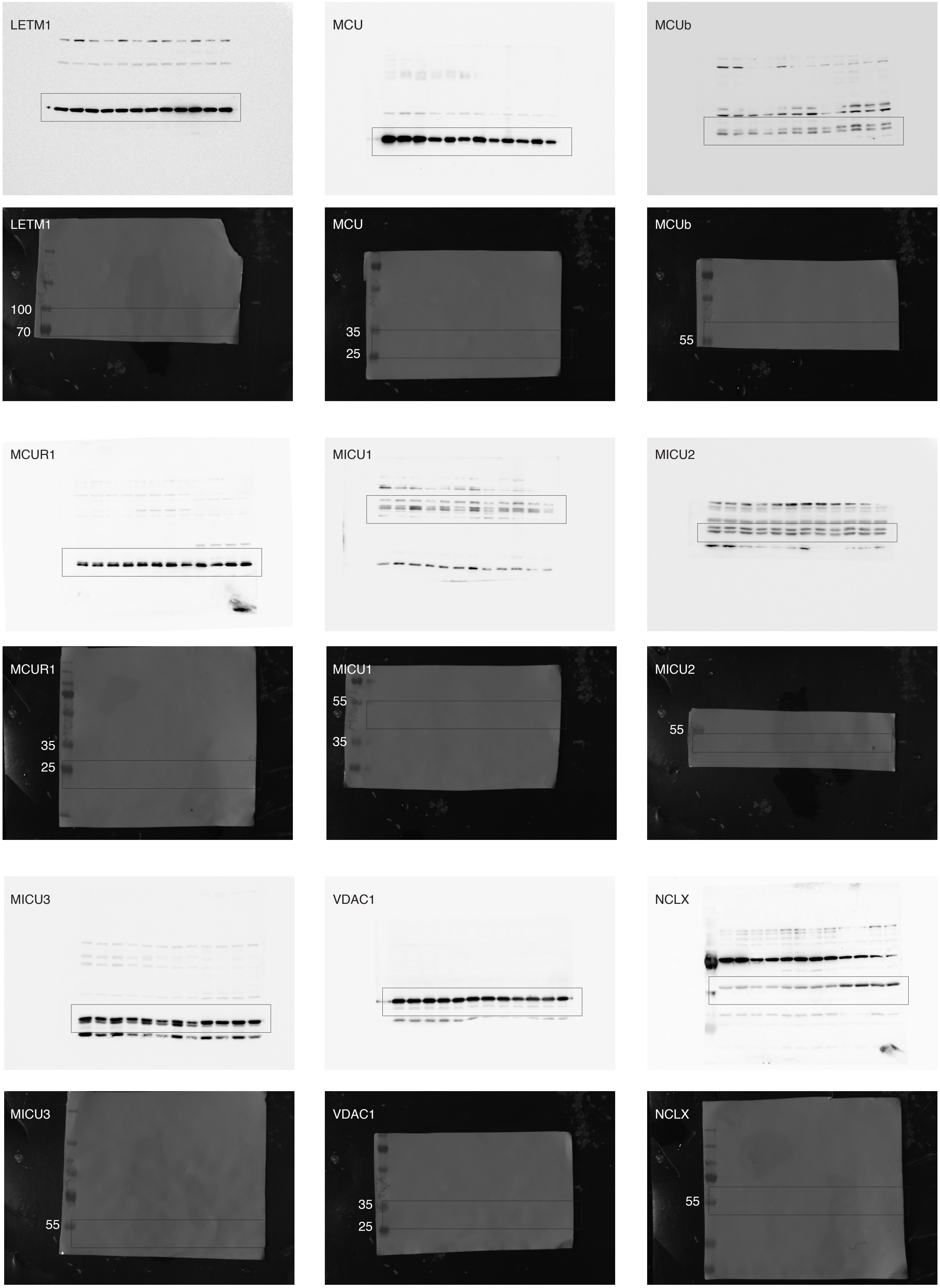

Fig. S6. Blot Transparency related to Supplementary Figure 1.

Journal of Cell Science • Supplementary information
