# Supplementary material for: Mitochondrial Ca2+ uniporter haploinsufficiency enhances long-term potentiation at hippocampal mossy fibre synapses
Source: J Cell Sci. 2022 Nov 23;135(22):jcs259823. doi: 10.1242/jcs.259823 (PMC10563808; doi:10.1242/jcs.259823)
Supplement: Supplementary file 1 [file joces-135-259823_review_history.pdf]

Mitochondrial Ca^{2+} uniporter haploinsufficiency enhances long-term potentiation at hippocampal mossy fibre synapses

Michael J. Devine, Blanka R. Szulc, Jack H. Howden, Guillermo López-Doménech, Arnaud Ruiz and Josef T. Kittler
DOI: 10.1242/jcs.259823

Editor: Giampietro Schiavo

Review timeline
Original submission: 28 January 2022
Editorial decision: 22 February 2022
First revision received: 6 October 2022
Accepted: 18 October 2022

Original submission

First decision letter

MS ID#: JOCES/2022/259823

MS TITLE: Mitochondrial Ca2+ Uniporter haploinsufficiency enhances long-term potentiation at hippocampal mossy fibre synapses

AUTHORS: Michael J Devine, Blanka Szulc, Jack Howden, Guillermo Lopez-Domenech, Arnaud Ruiz, and Josef Kittler
ARTICLE TYPE: Short Report

We have now reached a decision on the above manuscript.

To see the reviewers' reports and a copy of this decision letter, please go to: https://submit-jcs.biologists.org and click on the 'Manuscripts with Decisions' queue in the Author Area. (Corresponding author only has access to reviews.)

As you will see, the reviewers gave surportive reports but raised some critical points that will require amendments to your manuscript, which include addition of primary data. I hope that you will be able to carry these out because I would like to be able to accept your paper, depending on further comments from reviewers.

We are aware that you may be experiencing disruption to the normal running of your lab that makes experimental revisions challenging. If it would be helpful, we encourage you to contact us to discuss your revision in greater detail. Please send us a point-by-point response indicating where you are able to address concerns raised (either experimentally or by changes to the text) and where you will not be able to do so within the normal timeframe of a revision. We will then provide further guidance. Please also note that we are happy to extend revision timeframes as necessary.

Please ensure that you clearly highlight all changes made in the revised manuscript. Please avoid using 'Tracked changes' in Word files as these are lost in PDF conversion.

I should be grateful if you would also provide a point-by-point response detailing how you have dealt with the points raised by the reviewers in the 'Response to Reviewers' box. Please attend to

© 2022. Published by The Company of Biologists under the terms of the Creative Commons Attribution License (https://creativecommons.org/licenses/by/4.0/).

all of the reviewers' comments. If you do not agree with any of their criticisms or suggestions please explain clearly why this is so.

Reviewer 1

Advance summary and potential significance to field

Defining the role of mitochondrial calcium buffering at the synapse is timely and an important physiological/pathophysiological phenomenon that needs clarification.

Comments for the author

Big Picture Critique-
There are multiple interpretations for the findings that are unexplored. There is no discussion or demonstration of alterations that occur in other mitochondrial calcium handling proteins as a result of MCU haploinsufficiency or discussion of other possible ways by which calcium may enter the matrix (letm1) that may be affected by MCU insufficiency. Additionally, there is no functional demonstration of altered long term potentiation with any kind of behavioral study (Barnes maze probe trial, etc.). Additionally, the authors state that they can't do complete ablation of MCU because it's embryonic lethal (which is true on a global knockout only with specific inbred mouse strains). Complete knockout of MCU in neurons has been shown in the literature to not be embryonic lethal which would allow for both haploinsufficient and complete knockout groups and specificity with cell type. The most recent/relevant literature (which the author cited) was related to MICU3 lowering the threshold for mitochondrial calcium uptake in neuronal mitochondria and being responsible for activity driven ATP production at the presynaptic terminal.

Major concerns:
1. The Author does not discuss how MCU haploinsufficiency affects expression/activity of any other mitochondrial calcium handling machinery. It is well known that deletion of uniporter components results in changes in other regulatory subunits. The authors should blot for MICUs, MCUB, EMRE, NCLX, etc. (i.e., is this due to loss of MCU or remodeling of other calcium exchange proteins.) Perhaps not a big deal given the calcium data shown, but none the less gating should be examined.
2. The authors should directly compare the vGlutPhluorin data between WT mito and MCU-/+ mito. This is the important comparison for the hypothesis. Fig 2b, vs. 2d.
3. Figures 2g and 2i are confusing. Are the authors suggesting that the redistribution of mitochondria in response to PTX is MCU-dependent? If so again the wrong group comparisons are performed. WT vs. MCU+/- should be compared. Also, there looks to be an unequal distribution of cell responses in the DMSO MCU+/- group. Is this really different? Perhaps more cells should be analyzed with a direct comparison of MCU+/- vs. control or even better a second model with acute inhibition of Ru360 if this works in the authors' system.
4. The use of a model with global haploinsufficiency versus a glutaminergic-neuron specific MCU +/- leaves many confounding issues for interpretation. For example, glutamine clearance at the synapse is reliant on the astrocytic uptake of glutamine, ATP-dependent conversion to glutamate in the astrocyte, and recycled to the presynaptic terminal. MCU haploinsufficiency is likely to decrease ATP availability in the astrocyte and slow the cycle down possibly leading to increased glutamine in the synapse and altered neurotransmission and LTP. Also, using a glutaminergic specific cre would allow for complete knockout instead of just haploinsufficiency.
5. In wildtype mice, calcium peaks are much higher in boutons lacking mitochondria and the authors explain this by stating that the lack of MCU-
mediated Ca^{2+} clearance/buffering (2b). Figure 2d demonstrates that there is no difference in calcium clearance in boutons with mitochondria present versus those without mitochondria present in MCU +/- mice. Because these are only haplo-insufficient mice and not complete knockouts (i.e., you still have 50% of MCU protein), this refutes the overall conclusions of the paper. Are the mitochondria in the boutons of the MCU +/- mice clearing no calcium at all?
6. Experiments used to generate 3c and 3d to measure fibre volley should be repeated using chemical inhibitors to confirm that the observed affects are due to decreased MCU mediated Ca transport and not changes in sodium handling/other ion handling.

© 2022. Published by The Company of Biologists under the terms of the Creative Commons Attribution License (https://creativecommons.org/licenses/by/4.0/).

7. Since LTP and neurotransmitter release is also an energetic process, in addition to calcium signaling, the function of the uniporter in relationship to ATP bioavailability vs. calcium buffering should be explored.

Reviewer 2

Advance summary and potential significance to field

Mitochondria are a major calcium sink at the presynapse, therefore any modulation of their ability to accumulate calcium during neuronal activity is likely to impact neurotransmitter release and synaptic vesicle recycling. This has been demonstrated my multiple groups previously, however this study also extends this work to reveal effects on forms of plasticity that are dependent on the presynapse.

Comments for the author

This is a relatively simple paper exploring the role of the mitochondrial calcium uniporter (MCU) in calcium handing at the presynapse and the subsequent downstream effects on both synaptic vesicle fusion and neurotransmission. The work is well performed and explained. The findings are not particularly surprising in the context of the authors' previous research, however there is sufficient novelty with an extension of the work examining how increased presynaptic calcium due to MCU haploinsufficiency alters evoked neurotransmission and synaptic plasticity. I only have a few minor points to raise.
1) The statistical reporting in the figure legends could be improved by consistently providing the experimental n and the actual p value when a significant result is achieved. It would also be good to see the individual data points presented on all bar graphs. This is provided in some cases but not all.
2) Intracellular calcium also regulates SV endocytosis at the presynapse and modulation of MCU expression has small effects on the kinetics of this process (Marland et al (2016) JBC, 291:2080). It may be that haploinsufficiency of MCU is too mild to modulate SV endocytosis, however for completeness it would be interesting to confirm the presence or absence of effect by calculating the kinetics of the vGLUT-pHluorin fluorescence decrease (which reflects SV retrieval) in wild-type and heterozygous neurons.
3) In the context of the vGLUT-pHluorin studies, strictly speaking the fluorescence response reflects SV fusion and not neurotransmitter release (as referred to in the text). Obviously these events are sequential, however for accuracy it would be good to revert references to "neurotransmitter release" to either "SV fusion" or "SV exocytosis".
4) Page 9 – lines 13-14 "The magnitude of post-tetanic potentiation (PTP), which results from a slow efflux of tetanically accumulated mitochondrial Ca2+…". A reference would be good to cite here to support this claim.
5) Page 14 line 25 – DF/F0 should be "Greek DELTA"F/F0

First revision

Author response to reviewers' comments

Reviewer 1 Advance Summary and Potential Significance to Field...

Defining the role of mitochondrial calcium buffering at the synapse is timely and an important physiological/pathophysiological phenomenon that needs clarification.

Reviewer 1 Comments for the Author...

Big Picture Critique-
There are multiple interpretations for the findings that are unexplored.

© 2022. Published by The Company of Biologists under the terms of the Creative Commons Attribution License (https://creativecommons.org/licenses/by/4.0/).

There is no discussion or demonstration of alterations that occur in other mitochondrial calcium handling proteins as a result of MCU haploinsufficiency or discussion of other possible ways by which calcium may enter the matrix (letm1) that may be affected by MCU insufficiency.Additionally, there is no functional demonstration of altered long term potentiation with any kind of behavioral study (Barnes maze probe trial, etc.). Additionally, the authors state that they can't do complete ablation of MCU because it's embryonic lethal (which is true on a global knockout only with specific inbred mouse strains). Complete knockout of MCU in neurons has been shown in the literature to not be embryonic lethal, which would allow for both haploinsufficient and complete knockout groups and specificity with cell type. The most recent/relevant literature (which the author cited) was related to MICU3 lowering the threshold for mitochondrial calcium uptake in neuronal mitochondria and being responsible for activity driven ATP production at the presynaptic terminal.

We thank the reviewer for their comments. The reviewer raises an excellent point about whether there might be alterations in other mitochondrial calcium handling proteins resulting from MCU haploinsufficiency, and we have addressed this (new Supplementary Figure 1). However, we believe that behavioural studies, whilst certainly very interesting, are beyond the scope of this short report. Regarding the animal models used, the reviewer is absolutely correct that the impact of MCU knockout is strain dependent, and we have clarified this point in the manuscript (Methods; Animal models). Nevertheless, our data show that MCU+/- (rather than MCU-/-) is sufficient to impact mitochondrial regulation of presynaptic function.

Major concerns:

1. The Author does not discuss how MCU haploinsufficiency affects expression/activity of any other mitochondrial calcium handling machinery. It is well known that deletion of uniporter components results in changes in other regulatory subunits. The authors should blot for MICUs, MCUB, EMRE, NCLX, etc. (i.e., is this due to loss of MCU or remodeling of other calcium exchange proteins.) Perhaps not a big deal given the calcium data shown, but none the less gating should be examined.

We fully agree: we have carried out additional western blots on brain lysates from WT and MCU+/- mice probing for additional components of mitochondrial Ca2+ uptake machinery. These new data are shown in Supplementary Figure 1 (Results and Discussion, p. 5, lines 6- 9).

2. The authors should directly compare the vGlutPhluorin data between WT mito and MCU-/+ mito. This is the important comparison for the hypothesis. Fig 2b, vs. 2d.

In transfected neuronal cultures, as used in our study, vGlutpHluorin expression levels vary between neurons, which could mask differences in SV fusion quantity between neurons. Additionally, excitability varies between neurons in culture, so the vGlutpHluorin response will vary between neurons even with the same level of vGlutpHluorin expression. Therefore, we restricted our vGlutpHluorin analysis to comparing presynapes with and without mitochondria, within the same axon. In our opinion, this is the valid and meaningful comparison, and is the analysis approach that we used in Vaccaro et al. (PMID: 28039205). We have also added analysis of SV endocytosis kinetics, as per reviewer 2's request, since this analysis is not affected by vGlutpHluorin expression levels (Results and Discussion, p. 6, lines 5-9).

3. Figures 2g and 2i are confusing. Are the authors suggesting that the redistribution of mitochondria in response to PTX is MCU-dependent? If so, again the wrong group comparisons are performed. WT vs. MCU+/- should be compared.

We agree that Fig 2g and 2i could be misinterpreted. We have carried out this additional comparison:
PTX MCU+/- versus PTX control, two-tailed unpaired t-test = 0.0271 (significant).
This supports our interpretation that recruitment of mitochondria to presynapses, in response to PTX, is MCU-dependent. This is included in the Results section (p. 6, lines 27- 29).

Also, there looks to be an unequal distribution of cell responses in the DMSO MCU+/- group. Is this really different?

© 2022. Published by The Company of Biologists under the terms of the Creative Commons Attribution License (https://creativecommons.org/licenses/by/4.0/).

We have carried out this additional analysis: F test to compare variances between DMSO MCU+/- and DMSO WT groups, p value = 0.2357 (not significant). Therefore, the distribution of cell responses between these two groups is not significantly different. We have also compared DMSO MCU+/- versus DMSO control; two-tailed unpaired t-test = 0.389 (not significant). These additional analyses suggest that the two groups are not significantly different.

Perhaps more cells should be analyzed with a direct comparison of MCU+/- vs. control or even better a second model with acute inhibition of Ru360 if this works in the authors' system.

Given that the distribution of DMSO MCU+/- and DMSO control responses are not significantly different, we do not think that additional cells need to be analysed.

Regarding Ru360, whilst this is indeed a selective inhibitor of MCU, there are two problems with using it in cell culture. Firstly, it is rapidly oxidised in tens of minutes (Woods and Wilson. PMID: 31869674) therefore it would be difficult to use this to suppress MCU activity for prolonged periods (48 hours) as used in our study. Secondly, cell penetrance of Ru360 is highly variable (Abramov and Duchen. PMID: 18471431), which is particularly problematic in our study where we are analysing single cells. Both of these problems are overcome by using a genetic means to lower MCU activity, as used here.

4. The use of a model with global haploinsufficiency versus a glutaminergic-neuron specific MCU +/- leaves many confounding issues for interpretation. For example, glutamine clearance at the synapse is reliant on the astrocytic uptake of glutamine, ATP-dependent conversion to glutamate in the astrocyte, and recycled to the presynaptic terminal. MCU haploinsufficiency is likely to decrease ATP availability in the astrocyte and slow the cycle down, possibly leading to increased glutamine in the synapse and altered neurotransmission and LTP. Also, using a glutaminergic specific cre would allow for complete knockout instead of just haploinsufficiency.

For clarity, we performed our work in excitatory glutamatergic neurons that release glutamate from their presynaptic terminals, rather than glutaminergic neurons.

We think that the author here is referring to the glutamate-glutamine cycle, where glutamate released through synaptic vesicle exocytosis is taken up from the synapse by perisynaptic astrocytes through glutamate transporters, then transformed into glutamine, which can then be transported back to neurons to be converted back into glutamate for subsequent packaging into glutamatergic vesicles.

We agree that whether MCU+/- might impact this cycle is a very interesting possibility. However, our prediction would be that if MCU+/- decreases astrocyte ATP availability and slows the glutamate-glutamine cycle, this would <u>reduce</u> glutamate availability at presynapses in MCU+/- neurons, leading to impaired neurotransmission and LTP, since astroglial glutamine supply has recently been shown to be required to sustain physiological activity (Cheung et al. PMID: 35136061) as well as support increased synaptic activity (Tani et al. PMID: 24559677). This would be in contrast to what we observe in our experiments, which is <u>enhanced</u> neurotransmission and LTP in MCU+/- neurons.

5. In wildtype mice, calcium peaks are much higher in boutons lacking mitochondria and the authors explain this by stating that the lack of MCU-mediated Ca2+ clearance/buffering (2b). Figure 2d demonstrates that there is no difference in calcium clearance in boutons with mitochondria present versus those without mitochondria present in MCU +/- mice. Because these are only haplo-insufficient mice and not complete knockouts (i.e., you still have 50% of MCU protein), this refutes the overall conclusions of the paper. Are the mitochondria in the boutons of the MCU +/- mice clearing no calcium at all?

*We think the reviewer is referring to Figure **1g** rather than **2b**. Figure **1d** demonstrates that neuronal mitochondria in MCU+/- are still capable of mitochondrial Ca2+ uptake, albeit significantly less than mitochondria in WT neurons. This impairment in mitochondrial Ca2+ uptake is sufficient to block the effect of presynaptic mitochondria on buffering of presynaptic Ca2+ in MCU+/- neurons, as shown in Figure **1i**.*

© 2022. Published by The Company of Biologists under the terms of the Creative Commons Attribution License (https://creativecommons.org/licenses/by/4.0/).

6. Experiments used to generate 3c and 3d to measure fibre volley should be repeated using chemical inhibitors to confirm that the observed affects are due to decreased MCU mediated Ca transport and not changes in sodium handling/other ion handling.

We are not certain which chemical inhibitors the reviewer has in mind. Tetrodotoxin (Na+ channel blocker) would block the fibre volley, and therefore the experiments could not be done. Experiments using Ru360 (selective inhibitor of MCU) would be difficult to interpret for the same reasons given in response to point 3, i.e. rapid oxidation and highly variable cell penetrance. The chemical could in theory be added via patch pipette, but this approach would not be suitable for the electrophysiological experiments used in the study.

Fortunately these problems are circumvented by using a genetic means to lower MCU activity, along with avoiding any off target effects of chemical inhibitors.

7. Since LTP and neurotransmitter release is also an energetic process, in addition to calcium signaling, the function of the uniporter in relationship to ATP bioavailability vs. calcium buffering should be explored.

The reviewer is absolutely correct that there is a close relationship between Ca2+ signalling and ATP regulation/availability at presynapses. However, if MCU+/- were to significantly impact presynaptic ATP availability, we predict that this would lower ATP availabilty (due to reduced mitochondrial Ca2+ uptake). To examine this further, we have carried out additional experiments using the ratiometric ATP:ADP probe PercevalHR (Tantama et al. PMID 24096541) to determine ATP availability in MCU+/- neurons compared to wildtype controls. These new data are shown in a new Supplementary Figure 4 and are discussed in the Results section (p. 6, lines 10-15). As expected, there is less ATP in MCU+/- neurons at baseline, and following neuronal stimulation with glutamate, in keeping with mitochondrial Ca2+ uptake being a driver of mitochondrial ATP production.

Reviewer 2 Advance Summary and Potential Significance to Field...

Mitochondria are a major calcium sink at the presynapse, therefore any modulation of their ability to accumulate calcium during neuronal activity is likely to impact neurotransmitter release and synaptic vesicle recycling. This has been demonstrated my multiple groups previously, however this study also extends this work to reveal effects on forms of plasticity that are dependent on the presynapse.

Reviewer 2 Comments for the Author...

This is a relatively simple paper exploring the role of the mitochondrial calcium uniporter (MCU) in calcium handing at the presynapse and the subsequent downstream effects on both synaptic vesicle fusion and neurotransmission. The work is well performed and explained. The findings are not particularly surprising in the context of the authors' previous research, however there is sufficient novelty with an extension of the work examining how increased presynaptic calcium due to MCU haploinsufficiency alters evoked neurotransmission and synaptic plasticity. I only have a few minor points to raise.

We thank the reviewer for their positive comments on our report.

1) The statistical reporting in the figure legends could be improved by consistently providing the experimental n and the actual p value when a significant result is achieved. It would also be good to see the individual data points presented on all bar graphs. This is provided in some cases but not all.

We thank the reviewer for spotting these omissions. Experimental n and exact p values (wherever possible) have been added throughout, and individual data points have been added to bar graphs.

© 2022. Published by The Company of Biologists under the terms of the Creative Commons Attribution License (https://creativecommons.org/licenses/by/4.0/).

2) Intracellular calcium also regulates SV endocytosis at the presynapse, and modulation of MCU expression has small effects on the kinetics of this process (Marland et al (2016) JBC, 291:2080). It may be that haploinsufficiency of MCU is too mild to modulate SV endocytosis, however for completeness it would be interesting to confirm the presence or absence of effect by calculating the kinetics of the vGLUT-pHluorin fluorescence decrease (which reflects SV retrieval) in wild-type and heterozygous neurons.

This is an excellent point. Marland et al. showed that MCU knockdown accelerates SV endocytosis by reducing Ca2+ efflux from mitochondria following neuronal stimulation, therefore reducing the breaking effect of local Ca2+ on SV endocytosis.

Using the same approach as used by Marland et al., we have calculated tau (time constant) for SV endocytosis in MCU+/- and WT neurons:
MCU+/- tau = 15.72s (95% CI 13.6 to 18.39)
WT tau = 23.95s (95% CI 20.72 to 28.18)
F statistic 13.11 (p = 0.0003)
These tau figures are in close agreement with those reported in Marland et al.

So, MCU+/- is sufficient to enhance SV endocytosis kinetics. We have added this additional analysis to the manuscript (Supplementary Figure 3, Results and Discussion, p. 6, lines 5-9).

3) In the context of the vGLUT-pHluorin studies, strictly speaking the fluorescence response reflects SV fusion and not neurotransmitter release (as referred to in the text). Obviously these events are sequential, however for accuracy it would be good to revert references to “neurotransmitter release” to either “SV fusion” or “SV exocytosis”.

We thank the reviewer for highlighting our imprecise terminology, and we have made these text changes to the manuscript.

4) Page 9 – lines 13-14 “The magnitude of post-tetanic potentiation (PTP), which results from a slow efflux of tetanically accumulated mitochondrial Ca2+…”. A reference would be good to cite here to support this claim.

We agree that this should be referenced, and we have added relevant papers (Tang and Zucker. PMID: 9115741; Lee et al. PMID: 18077672).

5) Page 14 line 25 – DF/F0 should be "Greek DELTA"F/F0

We have corrected this typographical error.

<u>Second decision letter</u>

MS ID#: JOCES/2022/259823

MS TITLE: Mitochondrial Ca2+ Uniporter haploinsufficiency enhances long-term potentiation at hippocampal mossy fibre synapses

AUTHORS: Michael J Devine, Blanka R Szulc, Jack H Howden, Guillermo Lopez-Domenech, Arnaud Ruiz, and Josef T Kittler
ARTICLE TYPE: Short Report

I am happy to tell you that your manuscript has been accepted for publication in Journal of Cell Science, pending standard ethics checks.

© 2022. Published by The Company of Biologists under the terms of the Creative Commons Attribution License (https://creativecommons.org/licenses/by/4.0/).

Reviewer 1

Advance summary and potential significance to field

same as provided previously

Comments for the author

The new data are solid and answer many of my previous concerns. Congrats on a solid study. No further comments.

Reviewer 2

Advance summary and potential significance to field

As stated before: Mitochondria are a major calcium sink at the presynapse therefore any modulation of their ability to accumulate calcium during neuronal activity is likely to impact neurotransmitter release and synaptic vesicle recycling. This has been demonstrated my multiple groups previously, however this study also extends this work to reveal effects on forms of plasticity that are dependent on the presynapse.

Comments for the author

The authors have addressed the minor concerns that I raised, and I am happy to recommend acceptance.

© 2022. Published by The Company of Biologists under the terms of the Creative Commons Attribution License (https://creativecommons.org/licenses/by/4.0/).
